# Probing of breast cancer using a combination of plasma and urinary circulating cell-free DNA

**DOI:** 10.1042/BSR20194306

**Published:** 2020-11-03

**Authors:** Zhigang Zuo, Jiying Tang, Xiaojun Cai, Feng Ke, Zhenzong Shi

**Affiliations:** 1 Department of Oncology, Renmin Hospital, Hubei University of Medicine, Shiyan, Hubei 442000, China; 2 Department of Ophthalmology, Renmin Hospital, Hubei University of Medicine, Shiyan, Hubei 442000, China; 3 Department of Thyroid and Breast Surgery, Renmin Hospital, Hubei University of Medicine, Shiyan, Hubei 442000, China

**Keywords:** Cancer Relapse, Cell free DNA, Early breast cancer, Minimal residual disease

## Abstract

Monitoring of early-stage breast cancer is critical in promptly addressing disease relapse. Circulating cell-free DNA provides a minimally invasive and sensitive means to probing the disease. In a longitudinal analysis of 250 patients with early breast cancer, we compared the circulating cell-free DNA recovered from both plasma and urine specimens. For comparison, 50 healthy controls were also recruited. Specific mutations associated with the disease were profiled to determine the clinical sensitivity and specificity. Correlations of recovered concentrations of cell-free DNA with outcomes were examined to address early prognostication. PIK3CA mutation profiling in both plasma and urinary cell-free DNA showed an agreement of 97.2% compared with the results obtained for tumor tissues. The analysis of healthy controls revealed that cell-free DNA measurements were stable and consistent over time. Over the short 6-month period of monitoring, our analyses showed declines in recovered cell-free DNA; these findings may aid physicians in stratifying patients at higher risk for relapse. Similar results were observed in both plasma and urine specimens (hazard ratios: 2.16 and 2.48, respectively). Cell-free DNA presents a novel and sensitive method for the monitoring of early-stage breast cancer. In the present study, serial measurements of both plasma and urine specimens were useful in probing the disease.

## Introduction

Detection of early-stage breast cancer is important and closely linked to overall survival [1]. Owing to its global incidence [2], considerable attention is focused on early-stage breast cancer, and curative treatments are available [3]. Diagnosis is usually reached via radiographic imaging [4], and routine pre-screening of older and high-risk women has improved the detection of cases prior to cancer metastasis [5]. Metastatic recurrence in these patients is common [6], and may be partly due to minimal residual disease present after initial treatment [7]. The presence of these small tumor growths that may eventually lead to overt metastatic recurrence poses challenges to early detection and treatment.

Cell-free DNA in the systemic circulation originates from necrotic or apoptotic cells [8], and is subsequently present in urine due to the ultrafiltration process in the kidneys [9]. Tumor DNA has been detected in this specimen pool [10], showing clinical relevance to the disease. Probing of cancer using alternative means, such as blood sampling and urine testing, is an attractive prospect. Blood sampling and urine collection are minimally invasive and straightforward procedures for patients, and can be potentially used in the serial monitoring of patients with early-stage breast cancer. This may address the gaps in the detection of minimal residual disease, and provide opportunities for early interventions. The utility of cell-free DNA as a predictor of prognosis has been demonstrated in cancers of different origins [11], capturing the genetic heterogeneity of tumors [11], as well as the detection and treatment monitoring of advanced-stage non-small cell lung cancer [12].

The detection of early-stage breast cancer using cell-free DNA is challenging mainly due to the low concentrations of recovered material compared with studies examining advanced-stage cancers [13]. To ensure the accurate profiling of patients, we proposed to investigate the presence of cell-free DNA in both plasma and urine. Currently, there are limited data available for both plasma and urinary cell-free DNA in early-stage breast cancer. Technological innovations have led to the development of more sensitive detection assays, that may aid in addressing these deficiencies [14]. The aim of the present study was to assess the potential usefulness of cell-free DNA detection in probing and monitoring patients with early-stage breast cancer. Patients who underwent adjuvant systemic therapy and surgery were recruited into the trial. Pre- and post-treatment measurements of cell-free DNA from plasma and urine specimens were compared. We assessed the variations in the levels of cell-free DNA at different time intervals to identify potential correlations with disease relapse from patient follow-ups. The study provides a systematic means to assessing the levels of cell-free DNA, identifying its relevance in early-stage breast cancer. Moreover, it proposes the utility of cell-free DNA as a predictor of disease relapse that may complement current disease management processes.

## Methods

### Patient demographics and study design

All procedures, including the patient recruitment process and sample testing protocols, were approved by the institutional review board of Renmin Hospital (Shiyan, China). A total of 280 patients with confirmed early-stage breast cancer were recruited. A total of 30 patients had incomplete longitudinal data and were excluded from the analysis. Another 50 healthy donors were recruited as controls in the present study. Details of the trial participants are provided in Table 1. All participants provided informed consent and patient demographics were collected in the present study. Patients who underwent routine health screening or sought medical assistance due to suspicion of breast cancer were recruited from 2016 to 2018. Baseline measurements were performed prior to surgery, and followed up with monthly repeated blood and urine collections. Primary tissue biopsies were performed prior to treatment as part of the routine clinical investigation. Early-stage disease without signs of metastasis was confirmed in all patients using computed tomography. Healthy controls provided urine and blood specimens at monthly intervals for 6 months. Comparisons between genetic markers associated with the disease and changes in the levels of cell-free DNA post-treatment were performed. The estrogen receptor/progesterone receptor/human epidermal growth factor receptor 2 (HER2) status (Table 1) was determined as part of the routine investigation for patients with breast cancer.

**Table 1:** Early breast cancer patients’ and healthy volunteers’ demographics

	Cancer patients, *n*=250	Healthy volunteers *n*=50
Age Group		
<30	24	10
30–50	216	30
<50	10	10
Median Age		
	48	46
ER/PR/Her2 status		
HER2	113	NA
ER/PR+	48	NA
Triple negative	89	NA
Mutational Frequency		
PIK3CA	75	NA
Cancer Staging		
Stage I	198	NA
Stage II	52	NA

### Collection and preparation of blood and urine samples

Blood and urine specimens were collected on the same day to avoid discrepancy. Sampling was scheduled in morning visits during patient follow-ups. This approach ensured consistent sampling for all patients. Simultaneous collection of blood and urine specimens was performed. Peripheral blood (5 ml) was collected in an ethylenediaminetetraacetic acid blood tube by a trained phlebotomist. Approximately 50 ml of first, morning, mid-stream urine was collected. All samples were processed within 2 h. Plasma was extracted from whole blood through a two-step centrifugation process based on current laboratory practice. Blood was centrifuged at 3000 ***g*** for 10 min at room temperature, and the supernatant was carefully transferred to a new tube for repeated centrifugation. This ensured the effective removal of any cellular debris. The processing of urine was conducted using a similar two-step centrifugation process. Urine was transferred from the collection receptacle into a 50 ml centrifuge tube, and centrifuged at 5000 ***g*** for 10 min at 4°C. Subsequently, 30 ml of supernatant was transferred to another tube for repeated centrifugation.

### Purification and quantification of cell-free DNA

Purification of cell-free DNA was performed using the QIAamp cell-free Circulating Nucleic Acid Kit (Qiagen Inc., U.S.A.), according to the instructions provided by the manufacturer. The process was completed with multiple centrifugations using spin columns provided in the kit. Cell-free circulating DNA obtained from plasma and urine was eluted in 10 μl of tris-ethylenediaminetetraacetic acid (pH 8.0). Quantification of DNA was performed using the Nanodrop 2000 (Thermo Fisher Scientific, U.S.A.) with 1 μl of elute. To ascertain the clinical relevance and sensitivity of our assay, we attempted to detect a common mutation typically associated with breast cancer (PIK3CA) in the recovered cell-free DNA. These results were validated using the genetic profiles obtained from tissue biopsies. Briefly, DNA was extracted from the tissue specimens, and polymerase chain reaction (PCR) was used to amplify the entire coding regions of exons 1, 9 and 20. The amplicons were purified and sequenced through Sanger sequencing. These exons cover the majority of reported PIK3CA mutations associated with cancer. In the subsequent time course analysis, we assessed the consistency of detecting this mutation in both blood and urine samples. The detection of PIK3CA was performed using digital droplet PCR (ddPCR) for a sensitive readout. Commercially available primers and probes were purchased (BioRad Inc., U.S.A.), and the reaction was performed on the QX200 system (BioRad Inc.). Briefly, samples for ddPCR were batched, and the reaction was run in duplicate including internal positive and negative controls. Generation of droplets was accomplished using the QX200 droplet generator (BioRad Inc.) (approximately 14,000 droplets per reaction well). Amplification was performed under the following thermocycling conditions: 95°C for 10 min, followed by 40 cycles of 95°C for 15 s and 60°C for 60 s, 10 min at 98°C, and finally maintained at 4°C. Reaction plates were read on the QX200 reader, and the readout was generated by the Quantasoft software (BioRad Inc.).

### Statistical analysis

Data of the plasma and urine cell-free DNA levels and primary tumor tissue biopsies were tabulated and compared to ascertain the sensitivity and specificity of the assay. Student’s *t*-test was used to compare the levels of cell-free DNA obtained from plasma and urine samples. One-way analysis of variance (ANOVA) was conducted to compare the results at multiple time points. The coefficient of variations at different time points was determined to understand the stability of the marker. Kaplan–Meier analysis was performed to examine potential correlations with disease relapse. The hazard ratios (HRs) were computed to understand the influence of changes in cell-free DNA on disease outcomes.

## Results

### Baseline comparisons of cell-free DNA and tissue biopsies

**Figure 1:**
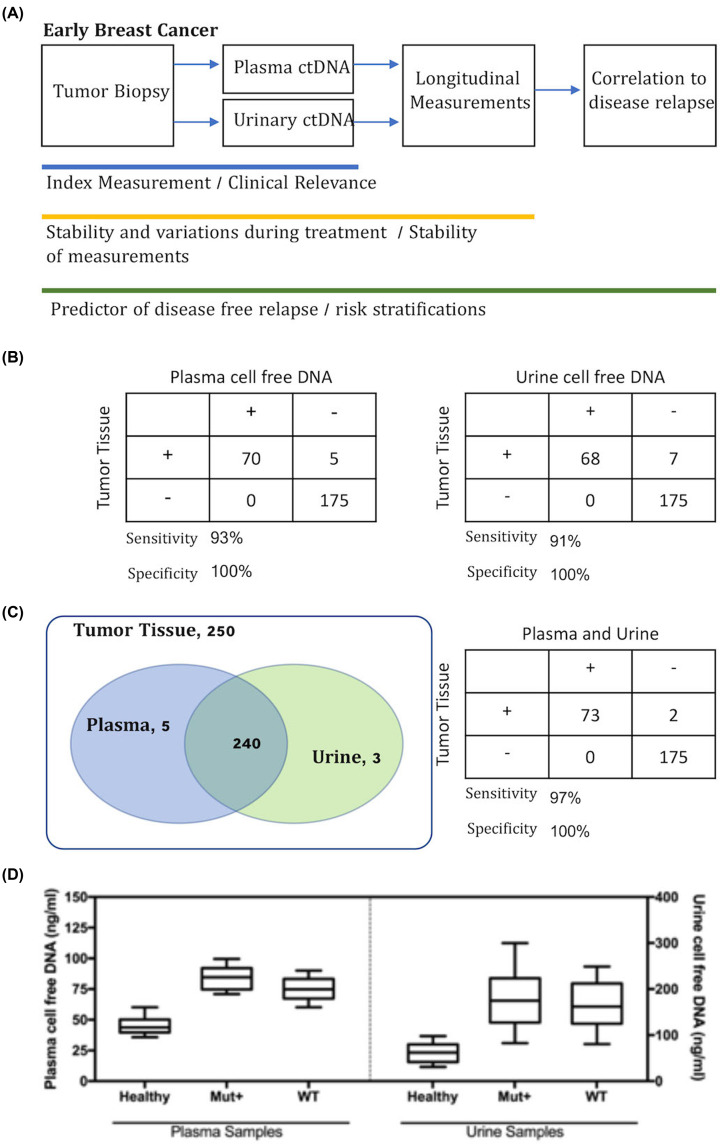
Study design and agreement with tumor tissues at baseline index measurements (**A**) Study design flow chart and measurement of plasma and urinary cell-free DNA. (**B**) Concordance of plasma and urine cell-free DNA with tumor tissues for PIK3CA. (**C**) Collative results of both plasma and urine samples in comparison with tumor tissue profiling showing strong overlap between the plasma and urine testing results. Sensitivity and specificity of plasma and urine testing were 97% and 100%, respectively. (**D**) Quantitative measurement of cell-free DNA in patients prior to treatment.

In the present study, we investigated the utility of cell-free DNA in early-stage breast cancer. The study also compared the use of plasma and urine cell-free DNA to assess the sensitivity and specificity of these assays (Figure 1A). As presented in Table 1, 250 patients and 50 healthy controls were serially monitored in the present study. The median age of patients with cancer and healthy controls was 48 and 46 years, respectively. All patients underwent surgery to remove the detected tumors. Index measurements refer to testing results of both urine and blood samples before surgical procedures. Tumor tissues were available for all patients with cancer, and the estrogen receptor/progesterone receptor and HER2 status was determined in these tissues as part of the routine clinical management. The proportions of patients harboring these mutations were 19.2% and 45.2%, respectively. Cell-free DNA extracted from healthy controls was tested using these assays, and exhibited wild-type characteristics. Additionally, a common point mutation of PIK3CA [15] was profiled in all samples. Overall, 2050 blood and urine specimens were tested, including seven and six measurements for each patient and healthy control, respectively.

At index measurement, we observed good agreement in PIK3CA detection between urine and plasma samples (Figure 1B). A 98% concordance of plasma cell-free DNA and tumor tissue samples was observed. Of note, there were no false positive results recorded. A similar level of agreement (97%) was reported for urinary DNA. Figure 1C shows the overall agreement of both assays with the tissue biopsies. For healthy controls, the results revealed a 100% wild-type. There was also a strong agreement among the plasma and urine testing results, with 240 samples showing identical findings. For both assays combined, the overall concordance was 99%, and the associated sensitivity and specificity of the cell-free DNA assays were 97% and 100%, respectively. We further compared the concentrations of recovered cell-free DNA from the plasma and urine samples. Figure 1D shows each individual recovery of cell-free DNA from the plasma and urine samples. Lower mean concentration of cell-free DNA was detected in both plasma and urine specimens of healthy controls. The dispersion of data points among the control population was limited compared with that noted in the patient cohort. The mean concentration of cell-free DNA detected in healthy controls was 45.2 ng/ml (standard deviation: 7.1 ng/ml). For patients with cancer, the mean recovered cell-free DNA from plasma was 1.72-fold higher than that obtained from controls (*P*<0.001). In urine samples, the mean recovered DNA from cancer patients was 2.73-fold higher than that extracted from controls (*P*<0.001).

### Longitudinal trending in plasma and urine cell-free DNA

**Figure 2:**
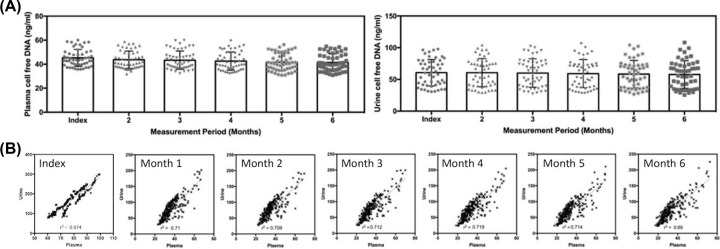
Serial trend monitoring of cell-free DNA using plasma and urine samples (**A**) Stability of results for plasma and urine samples over a 6-month period for healthy volunteers. (**B**) Correlation of plasma and urine cell-free DNA in patients with breast cancer over a period of 6 months that demonstrated good correlation among the two types of samples.

Serial samplings from patients with cancer were performed for 6 months post-surgery. In healthy controls, six measurements were performed at monthly intervals to address the stability of the assay, and provide a comparison for plasma and urine cell-free DNA. Figure 2A shows the results of serial measurements in healthy controls. ANOVA did not reveal significant changes in the plasma and urine cell-free DNA of healthy individuals. The coefficient of variation within the control cohort was similar at different measurement time points. For patients with cancer, we performed a direct comparison of plasma and urine cell-free DNA recovered at different time points, as shown in Figure 2B. We observed good linearity among the results of both assay, and the linear correlation coefficient demonstrated a very strong association. Coupled with the results of the PIK3CA profiling, both plasma and urine samples showed excellent correlations. Among the different time points, the index measurement showed the best correlation (*r*=0.874), while the measurement post-surgery showed the highest variability (*r*=0.690).

**Figure 3:**
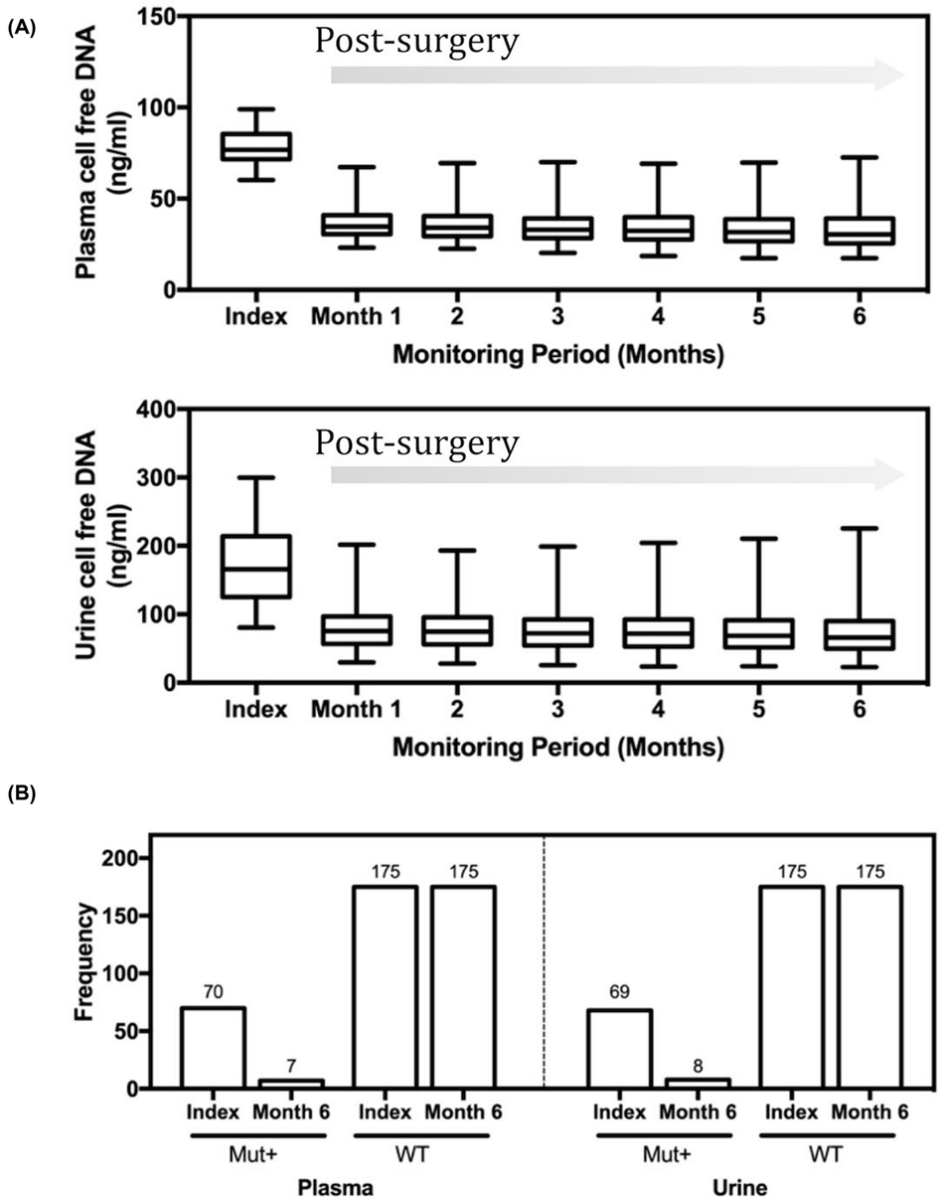
Trends of cell-free DNA in plasma and urine specimens after treatment (**A**) Significant declines in cell-free DNA were observed post surgery for both plasma and urine measurements. (**B**) Concordance of PIK3CA comparison at index (baseline) and the end of monitoring period showing 100% wild-type (WT) agreement, and approximately 10% of mutation-positive patients with persistent outcomes.

In addressing the trends of cell-free DNA post-surgery, we observed a marked decrease in both plasma and urine cell-free DNA (Figure 3A). A number of patients also exhibited reduction in the levels within the 95% confidence interval range of the healthy controls at baseline. For plasma samples, there was a 2.12-fold decrease in mean value compared with the index measurement for patients (*P*<0.001). For urine samples, there was a 2.10-fold decrease compared with the index results (*P*<0.001). The variability among patients was significant for both plasma and urine cell-free DNA. The ratio of maximum-to-minimum reduction among patients with breast cancer for plasma and urine cell-free DNA was 3.04 and 3.13, respectively. At the end of the 6-month monitoring period, PIK3CA analysis was performed on samples obtained from patients whose initial biopsy specimens showed positive mutation results. Interestingly, of the original 73 samples detected at the index measurement for urine and plasma testing, 11% remained positive (Figure 3B). The good agreement among urine and plasma samples in cell-free DNA highlights the close association of both sample types. The observed trends and characteristics may indicate different responses to treatment, and may further correlate to disease relapse.

### Correlation of disease relapse in patients with different levels of cell-free DNA

**Figure 4:**
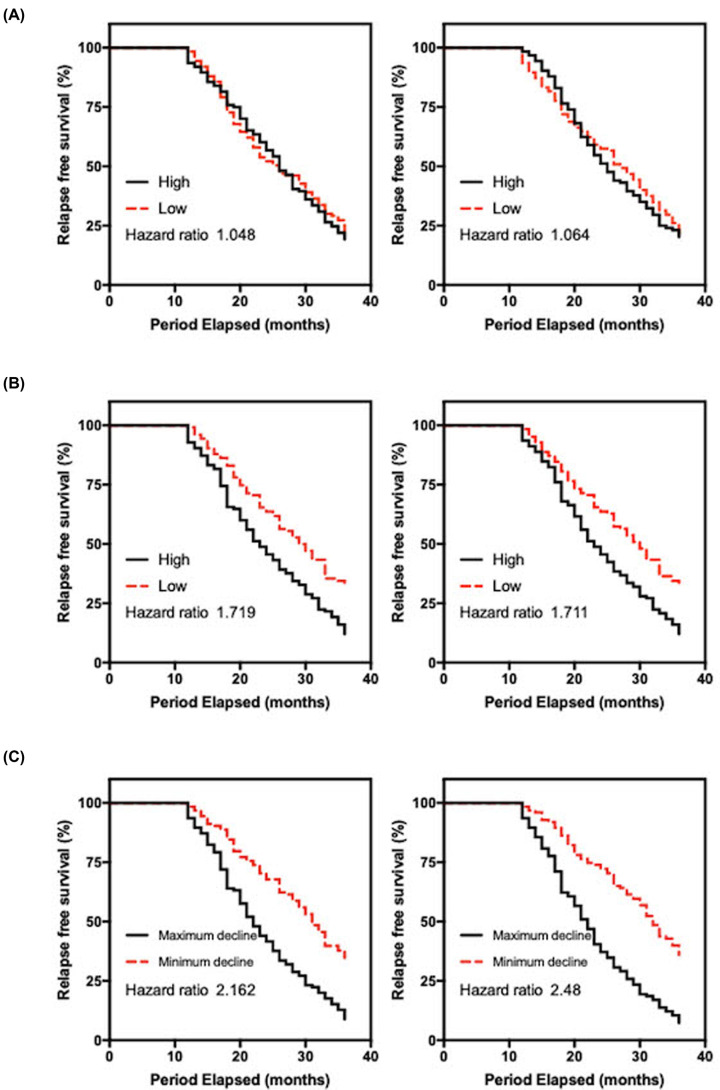
Relapse analysis of patients with early-stage breast cancer (**A**) Patients with breast cancer were divided equally based on the levels of cell-free DNA before treatment. The hazard ratio (HR) for plasma and urine testing was 1.05 and 1.06, respectively. (**B**) Comparison of patient subgroups divided equally based on the levels of cell-free DNA at 6 months. The HR for plasma and urine testing was 1.72 and 1.71, respectively. (**C**) Patients with breast cancer divided according to the decrease in cell-free DNA in plasma and urine. A median division of the patient population was selected. The HR for plasma and urine testing was 2.16 and 2.48, respectively, and showed the best correlation among all other measures.

The interesting trends observed in individual patients through serial monitoring highlight the possibility of varying treatment responses. Patients were monitored during routine patient follow-up visits or via phone calls at 3-month intervals. We investigated whether patients with higher levels of recovered cell-free DNA or least significant declines compared with index results were more susceptible to relapse. As indicated in Figure 4A, the index measurement for both plasma and urine cell-free DNA showed a slightly higher HR for the patient group associated with higher DNA levels. The patient cohort was divided equally based on the median recovered cell-free DNA. The HR for the plasma and urine analyses was 1.05 (95% confidence interval [CI]: 0.79–1.40) and 1.06 (95% CI: 0.80–1.42), respectively. We repeated the analyses at the end of the monitoring period as indicated in Figure 4B. Interestingly, the results showed higher HR in both analyses. For plasma cell-free DNA, the patient group with higher recovered content was linked to worse outcome and had an HR of 1.72 (95% CI: 1.29–2.29). Comparatively, the urine sample analysis showed similar trends with an HR value of 1.71 (95% CI: 1.28–2.28). Our data charting differences pre- and post-surgery showed different trends, which may be related to treatment responses. In a similar analysis, we separated the patient population according to the degree of decline in urine and plasma cell-free DNA. Figure 4C shows the results of the comparison. In a number of cases, the maximum declines were observed a few months post surgery. For plasma DNA, patients with greater declines were less susceptible to relapse within the 3-year follow-up period (HR: 2.16; 95% CI: 1.62–2.89). A similar result was observed urinary DNA (HR: 2.48; 95% CI: 1.85–3.33). Overall, we observed that urinary DNA may be slightly better for risk stratification in this setting.

## Discussion

The risk of disease relapse in patients with early-stage breast cancer is significant and, in part, may be attributed to missed micrometastases during diagnosis [16]. This highlights the need for more thorough monitoring of patients following the surgical removal of cancerous tissues. In the present study, we utilized a minimally invasive method to probe patients with early-stage breast cancer. The extraction of cell-free DNA from plasma and urine using currently available laboratory techniques is a straightforward process. In addition, patients are compliant with blood sampling and urine collection, thereby allowing repeated sampling. In several earlier studies, plasma cell-free DNA showed important applications in oncology [11–13,17]. From the discovery of molecular targets to unravelling therapeutic resistance in breast cancer [13], detection of cell-free DNA coupled with newer technologies in genomic sequencing has allowed a finer examination of genetic aberrations. For urinary cell-free DNA, much remains to be investigated and correlated to plasma DNA. In the present study, the utility of both plasma and urinary cell-free DNA was investigated. The main challenge in this patient cohort was the limited quantity of cell-free DNA collected from patients with early-stage breast cancer.

In our comparison of both plasma and urine results, we observed a very strong correlation. The correlation coefficient calculated from the direct comparison of recovered plasma and urine cell-free DNA showed values >0.9 in all cases. Similar results were obtained from the comparison with matched tumor biopsy samples, where the overall concordance rates were >0.9. The need for new tools to probe breast cancer will allow a better understanding of the disease pathogenesis. Complementing the proposed assays with routine testing via conventional imaging techniques will allow better detection of disease relapse. An earlier study conducted by Lipson et al., suggested that the levels of plasma cell-free DNA were indicative of tumor burden in advanced-stage disease [17]. The study also demonstrated that monitoring of these markers post treatment provides a better understanding of its real-time changes. We build upon these results to achieve short-term monitoring using plasma and urinary cell-free DNA. At baseline measurement, there was a statistically significant difference in the mean concentration of cell-free DNA detected in patients with early-stage breast cancer versus healthy controls. This different trend observed may be of clinical relevance. PIK3CA profiling showed high clinical specificity and sensitivity in using plasma and urine specimens compared with tumor tissues. This may be useful in supplementing current testing methodologies, and confirming key driver mutations for certain groups of patients. Such procedures will be useful in a number of cases; for example, patients for whom good biopsy samples cannot be retrieved, and possibly the geriatric population who are more resistant to undergoing surgical procedures for diagnostic investigations. Our results indicated that urinary cell-free DNA can be equally effective and can be used in tandem to plasma cell-free DNA for confirmation.

Subsequent serial monitoring demonstrated the utility of this approach for the detection of disease relapse. A key benefit of such assays is the ease of repeated measurements without causing significant discomfort to patients. We observed several interesting trends related to the monitoring of these patients over a 6-month period. A significant decrease in both urinary and plasma cell-free DNA post surgery reflected the intervention performed. Varying results were observed among these patients, notably in the declines of the levels of cell-free DNA post surgery. Given the close association of cancer with the levels of cell-free DNA, we performed a Kaplan–Meier analysis to address disease relapse. In the molecular profiling of patients after 6 months, we continued to detect mutations related to the primary tumor that may be linked to clinical outcome. Indeed, as shown in the present study, patients with a larger decline in cell-free DNA levels had better observed outcomes in terms of disease-free survival. The persistence and occurrence of higher levels of cell-free DNA that may have disease implications highlights the deficiencies in current management routines. Urinary cell-free DNA performed slightly better in risk stratification in this cohort. This needs to be further explored in studies with larger sample sizes. Moreover, a better understanding of the constituents of the nucleic acid content through deep genetic sequencing is desired. The usefulness of baseline data for risk prediction seems to be limited, possibly due to treatment responses that were not taken into consideration. This was previously demonstrated in a study conducted by Garcia-Murillas et al. [18]. Consistent with our findings, the results showed that plasma cell-free DNA was not a predictor of disease relapse. Better tools that will allow easier and more frequent probing of patients are currently available [19]. A limitation of the present study is that the levels of the recovered DNA were low. The disease is heterogenous and the detection of various cancer-associated mutations is challenging. Detecting these key driver mutations and oncogenes can aid in further increasing the sensitivity of prognostication. It will be of value to process larger number of samples and utilize novel methods [20] to make better use of tumor materials. Our study demonstrated the effectiveness and usefulness of cell-free DNA obtained from both plasma and urine samples. Additional routine probing is required to detect relapse in patients with early-stage breast cancer.

In conclusion, our study aims to provide a systematic understanding of cell-free DNA in early-stage breast cancer. We investigated the possibilities of using plasma and urinary cell-free DNA for the detection of mutation, disease monitoring, and prediction of disease relapse. Disease relapse in patients with breast cancer is a serious concern, and these patients will benefit from more frequent testing. The comparison of both plasma and urinary cell-free DNA yielded similar characteristics, results and trends. For the profiling of PIK3CA mutation, the combination of both assays increased the ability to detect positive samples. Comparing both sample types, urine sampling may also provide an easy method for sample collection, that may be more attractive to the geriatric population.

## Data Availability

The data sets used and/or analyzed during the current study are available from the corresponding author on reasonable request.

## Abbreviations

CI: confidence interval
ddPCR: digital droplet PCR
PCR: polymerase chain reaction
